# Intuitive moral bias favors the religiously faithful

**DOI:** 10.1038/s41598-024-67960-4

**Published:** 2024-08-07

**Authors:** Alex Dayer, Chanuwas Aswamenakul, Matthew A. Turner, Scott Nicolay, Emily Wang, Katherine Shurik, Colin Holbrook

**Affiliations:** 1 Department of Cognitive and Information Sciences, University of California, 5200 Lake Road, Merced, CA 95340 USA; 2 Interdisciplinary Humanities Graduate Group, University of California, 5200 Lake Road, CA Merced, USA

**Keywords:** Psychology, Human behaviour

## Abstract

Belief in powerful supernatural agents that enforce moral norms has been theoretically linked with cooperative altruism and prosociality. Correspondingly, prior research reveals an implicit association between atheism and extreme antisociality (e.g., serial murder). However, findings centered on associations between lack of faith and moral transgression do not directly address the hypothesized conceptual association between religious belief and prosociality. Accordingly, we conducted two pre-registered experiments depicting a “serial helper” to assess biases related to extraordinary helpfulness, mirroring designs depicting a serial killer used in prior cross-cultural work. In both a predominantly religious society (the U.S., Study 1) and a predominantly secular society (New Zealand, Study 2), we successfully replicated previous research linking atheism with transgression, and obtained evidence for a substantially stronger conceptual association between religiosity and virtue. The results suggest that stereotypes linking religiosity with prosociality are both real and global in scale.

## Introduction

Belief in supernatural agents that monitor and enforce moral conduct is broadly associated with prosociality^1–3^, including inclinations to punish those who fail to cooperate^4,5^, theoretically reinforcing prosocial tendencies. Religious doctrines often include prosocial rules^6,7^ that provide consistent and enforceable community norms regarding equitable behavior and expectations of mutual aid from others, including co-religionist strangers^8,9^. Consonant with the hypothesis that shared religious identities might enhance trust between in-group strangers^10^, researchers have observed that religious individuals invest significantly more trust in other religious individuals relative to non-believers^11^. Accordingly, societies characterized by religious beliefs in moralizing gods may have enjoyed greater capacities for large-scale trust and cooperation^12^, and the pervasiveness of the tendency to intuitively link religious faith with prosociality may reflect the cultural evolution of religion^13^.

Whether or not cultural group selection indeed favored religious societies due to enhanced cooperation, non-believers are liable to be perceived as morally dubious relative to believers to the extent that religiosity connotes prosocial and trustworthy qualities. That is, religious individuals are likely to be psychologically stereotyped as being more prosocial than non-religious individuals. For example, researchers found that individuals judged several extreme, counternormative acts (serial murder, incest, necrobestiality, and cannibalism) as more representative of atheists compared to other social and religious groups^14^, and convergent research indicates that distrust of atheists is mediated by the belief that supernatural surveillance encourages moral behavior^15^. In a complementary finding, Gervais et al.^16^ found that atheists are perceived to be less warm, competent, and trustworthy compared to theists, while more likely to commit immoral acts. Similarly, other research found that atheists were more likely to be perceived as prone to infidelity than religious individuals^17^. Perhaps unsurprisingly given the above findings, Americans find atheism to be the most unfavorable religious category^18^. While it is unclear if religious individuals are actually more prosocial than non-religious individuals, stereotypes of atheists as being less prosocial than religious individuals appear to be widespread.

Gervais et al.^19^ provided consistent evidence that extreme moral bias against atheists is a global phenomenon detectable even in relatively secular societies. Participants in thirteen societies read a vignette describing a person who tortures animals as a child and eventually goes on to murder five homeless people, then were asked to judge whether it was more probable that the person in the vignette was a teacher, or, in a between-subjects manipulation, a teacher framed either as a religious believer or as an atheist. The dependent variable of interest was the relative commission of the *conjunction fallacy* when comparing religious and atheist targets. The conjunction fallacy occurs when individuals violate the conjunction rule: the probability of “X and Y” being true cannot logically be greater than the probability of X alone being true^20^. Here, the ratio of participants selecting a subtype of teacher (either ‘a teacher who believes in God’ or ‘a teacher who does not believe in God’) rather than the logically correct choice of ‘teacher’ theoretically reflects an intuitive association with extreme moral transgression. Participants across all but two predominantly secular societies (Finland and New Zealand) were more likely to select the conjunction of teacher-and-atheist than teacher-and-believer.

Although Gervais et al.^19^ found that moral bias against atheists is both real and global in scope, they did not investigate how religiousness may be associated with stereotypes of religious individuals’ tendency to engage in morally praiseworthy actions. If religious belief spurs cooperation, this implies a positive conceptual association with religiousness and prosociality, not just a negative association between religious belief and transgressive acts such as murder. Moreover, the results of Gervais et al.^19^ might conceivably be explicable in terms of an association between the relatively atypical social trait of atheism and taboo, criminal, or otherwise socially counternormative behavior for reasons orthogonal to morality. This interpretation would be ruled out if atheists were found not to be conceptually associated with an atypical behavior. The present research addresses both limitations by testing for intuitive conceptual link between religiosity and atypical prosociality.

In Study 1, we modified the methods of Gervais et al.^19^ to test whether religious belief is intuitively linked with extreme prosociality, as well as attempt to replicate their previous U.S. findings linking atheism with serial murder. Mirroring the vignette describing a serial killer who murders homeless individuals, we created a vignette describing a serial helper who provides an extraordinary degree of altruistic assistance to homeless individuals. Additionally, we investigated the extent to which individual differences in degree of belief in God, which were mildly predictive of conjunction fallacy rates in Gervais et al.^19^, influence conjunction fallacy rates when considering a serial helper.

## Study 1

### Method

All studies were approved by the University of California, Merced, Institutional Review Board and performed in accordance with guidelines governing research with human participants. Informed consent was obtained from each participant.

#### Participants

Following Gervais et al.^19^, we recruited U.S. participants using Amazon’s Mechanical Turk. Following the recommended practice of increasing the initial sample size when conducting replication studies^21^, we sought to double the size of the U.S. sample of the original study (198 participants) for the replication (the serial killer conditions), as well as to obtain a comparable sample size for the novel serial helper conditions. As we expected the exclusion rate to resemble that of the original study (approximately 13% of responses), we recruited an additional 26 participants per condition, for a total of 904 participants. We restricted the study to individuals who had completed at least 500 prior tasks with a 99% or greater approval rate. Following our pre-registered exclusion criteria, we filtered responses that came from identical IP addresses, were incomplete, or failed an attention check. This filter yielded a rejection rate of 18%, leaving 744 valid responses (43.5% female; *M*_Belief in God_ = 48.28, *SD* = 41.77). Participants were paid $0.60 upon completion of the study.

#### Design

Extending the design of Gervais et al.^19^, half of our participants were randomly assigned to a prosocial condition in which they were asked to read a vignette that describes a man who helps animals as a child and then eventually becomes a regular helper of homeless individuals. After reading the vignette, participants were asked to judge whether it is more probable that the helper is a teacher, or, in a between-subjects manipulation, a teacher who either “does believe in God” or “does not believe in God”. Directly replicating the design of Gervais et al.^19^, half of our participants were randomly assigned to the same task, but referring to a man who tortures animals as a child and then eventually murders homeless people. Individuals in the serial killer conditions read the following vignette:

“When a man was young, he began inflicting harm on animals. It started with just pulling the wings off flies, but eventually progressed to torturing stray cats and other animals in his neighborhood.

As an adult, the man found that he did not get much thrill from harming animals, so he began hurting people instead. He has killed 5 homeless people that he abducted from poor neighborhoods in his home city. Their dismembered bodies are currently buried in his basement.

Which is more probable?

(A) The man is a teacher.
(B) The man is a teacher and does [does not] believe in God.”

Individuals in the serial helper conditions read the following vignette:

“When a man was young, he began helping stray animals. It started with just putting out water for birds, but eventually progressed to fostering stray cats and other animals in his neighborhood.

As an adult, the man felt inspired to help people in need as well. Each week he visits poor neighborhoods in his city and offers food and clothes to homeless people. Sometimes when the weather is very cold he offers homeless families a place to stay in a spare room in his house.

Which is more probable?

(A) The man is a teacher
(B) The man is a teacher and does [does not] believe in God.”

Participants then completed a series of distractor items (e.g., logical and mathematical puzzles), as in Gervais et al.^19^, before encountering demographic questions, including measures of belief in God and subjective socioeconomic status (SSES, a covariate included in Gervais et al.^19^). SSES was assessed according to an 11-point scale (0 = *Bottom*; 10 = *Top*) using a modified version of the MacArthur Scale of Subjective Social Status^22^. Belief in God was measured on a linear scale from 0 to 100 using the same single-item measure employed by Gervais et al.^19^:

“How strongly do you believe in God or gods (from 0–100)? To clarify, if you are certain that God (or gods) does not exist, please put "0" and if you are certain that God (or gods) does exist, then put "100).”

Upon completion of the study, participants were thanked, debriefed, and compensated. (We also collected an exploratory measure of covarying political orientation, inclusion of which does not change the pattern of results, and an exploratory measure of the degree to which participants subjectively felt themselves to be spiritually connected with God; see Supplemental Online Materials [SOM]).

Following Gervais et al.^19^, we analyzed the data using Bayesian logistic regression modeling and the *rethinking* package in R. The Bayesian estimation employed here allowed us to estimate uncertainty using highest posterior density intervals (HPDIs), which is a range of values that contains the most credible parameter values.

### Results

#### Target type (religious versus atheist) and the relative risk of the conjunction fallacy in the Serial Helper condition

We fit a logistic regression where the probability in the logistic function is a linear function of several explanatory variables: target type (religious or atheist), self-reported belief in God, age, gender, and self-reported subjective socioeconomic status. We also included interaction terms between target type and belief in God (see Table 1).

**Table 1:** Full Model Summary for the Serial Helper Condition (U.S., Study 1).

Fixed effects	Coefficient	SD	95% HPDI	
			2.5%	97.5%
Target: Religious vs. Atheist	− 3.73	0.48	− 4.76	− 2.88
Belief in God	0.91	0.17	0.59	1.25
Age	0.15	0.15	− 0.14	0.45
Gender	− 0.12	0.14	− 0.40	0.16
SSES	0.13	0.14	− 0.15	0.40
Target X Belief	− 2.19	0.45	− 3.18	− 1.37

Age, gender, subjective socioeconomic status (SSES) and participant belief in God were standardized. Religious vs. atheist target was coded: atheist = 1, religious = 0. The 95% highest posterior density intervals (HPDI) illustrate uncertainty around posterior means, and indexes the interval in which the 95% most credible estimates lie.

**Figure 1:**
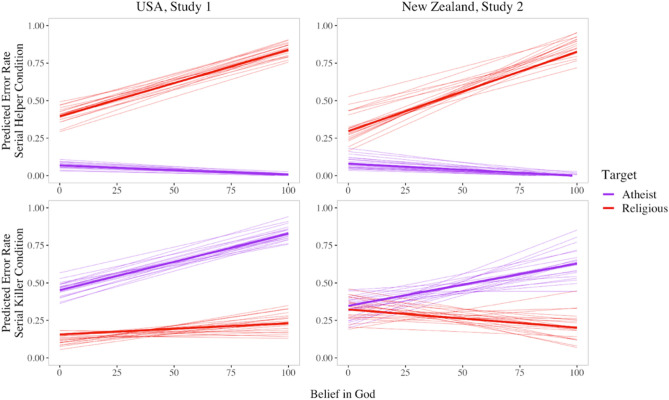
Summary of results from Study 1 (US) and Study 2 (NZ). *Note.* Predicted effect of participant belief in God, adjusting for individual gender, age and subjective socioeconomic status. Bold lines are overall estimates; thin lines display 20 predictions constructed from a set of parameters sampled randomly from the posterior to depict estimate uncertainty. Participants in the US (Study 1, left panels) and New Zealand (Study 2, right panels) were asked if the Serial Helper (top panels) or Serial Killer (bottom panels) was either a teacher or a teacher who was religious [an atheist]. The predicted conjunction fallacy error rates for different target types as a function of belief in God are visualized above.

Consistent with predictions, the conjunction fallacy was significantly more likely to be committed when the target was framed as religious than as an atheist. The overall conjunction fallacy error rate probability was only 4% for atheist targets (95% HPDI 0.01, 0.70), but 60% for religious targets (95% HPDI 0.52, 0.68). The probability of making the conjunction fallacy error was 19.4 times greater when the target was framed as religious. The full model likewise showed a significant effect of target type, as well as a main effect of belief in God qualified by a significant interaction with target type, such that participants who reported higher levels of belief in God were more likely to commit the conjunction fallacy in the religious target condition (see Fig. 1, top left panel).

#### Target type (religious versus atheist) and the relative risk of the conjunction fallacy in the Serial Killer condition

Replicating the findings of Gervais et al.^19^, the conjunction fallacy was significantly more likely to be committed when the target was framed as an atheist than as religious. The model predicted an overall conjunction fallacy error rate probability of 64% for atheist targets (95% HPDI 0.56, 0.72) compared to 18% for religious targets (95% HPDI 0.13, 0.24). The relative risk of making the conjunction fallacy error was nearly 3.6 times greater when the target was an atheist (the relative risk having been 2.5 times greater in the U.S. sample of Gervais et al.^19^ original study’s U.S. sample). The full model likewise showed a significant effect of target type (see Table 2). Departing from the results of prior work of Gervais et al.^19^, there was a significant interaction between target type and belief in God, such that higher levels of belief in God predicted greater conjunction fallacy rates for atheist targets than religious targets (see Fig. 1, bottom left panel).

**Table 2:** Full Model Summary for the Serial Killer Condition (U.S., Study 1).

Fixed effects	Coefficient	SD	95% HPDI	
			2.5%	97.5%
Target: Religious vs. Atheist	2.02	0.25	1.53	2.52
Belief in God	0.18	0.19	− 0.19	0.56
Age	− 0.02	0.13	− 0.27	0.23
Gender	− 0.07	0.13	− 0.32	0.18
SSES	− 0.13	0.13	− 0.38	0.12
Target X Belief	0.63	0.25	0.14	1.14

Age, gender, subjective socioeconomic status (SSES) and participant belief in God were standardized. Religious vs. atheist target was coded: atheist = 1, religious = 0. The 95% HPDI illustrates uncertainty around posterior means and indexes the interval in which the 95% most credible estimates lie.

### Discussion

In Study 1, we not only replicated Gervais et al.^19^, finding that the conjunction fallacy was more likely when a serial killer target character was framed as an atheist, but we also found a notably larger tendency to commit the conjunction fallacy when an altruistic serial helper character was framed as religious. Together, these results support the hypothesis that religiosity is broadly perceived as a cue that an unknown individual is not merely unlikely to commit moral transgressions, but disposed to engage in morally praiseworthy, prosocial acts, and hence a trustworthy cooperation partner. As in Gervais et al.^19^, we found conceptual associations between religious belief and morality in both the serial helper and serial killer conditions even among participants who reported low levels of religious belief. Nonetheless, we also observed significant interactions such that participants who reported greater religious faith were more prone to associate both the character framed as a believer with prosociality and the character framed as a nonbeliever with antisociality. These results also held when including political orientation and spiritual connection to God as predictors, and also when omitting all demographic variables.

The present findings suggest that religiosity and benevolence are asymmetrically conceptually linked relative to atheism and malevolence. Accordingly, whereas Gervais et al.^19^ did not find evidence of intuitive moral prejudice against atheists in the mostly secular societies of Finland and New Zealand, the possibility remains that a bias favoring the faithful as prosocial may be found within such societies. To test this possibility while allowing for direct comparison with the U.S. sample of Study 1, we next replicated the same design in the English-speaking, relatively secular nation of New Zealand.

## Study 2

### Method

#### Participants

We recruited participants from New Zealand using the service Prolific. We sought a sample of 600 participants, which, when accounting for the addition of the serial helper condition, would have been comparable to double the sample size per cell of the New Zealand sample of 161 participants used in the conceptually analogous study conducted by Gervais et al.^19^; however, we were only able to recruit 376 participants. We filtered responses as in Study 1, yielding a rejection rate of 4.7%, leaving 358 valid responses (52% female; *M*_Belief in God_ = 36.59; *S D* = 37.82). Participants were paid $1.00 upon completion of the study. Informed consent was obtained from each participant.

#### Design

The design was identical to that of Study 1.

### Results

The data were modeled as in Study 1.

#### Target type (religious versus atheist) and the relative risk of the conjunction fallacy in the Serial Helper condition

The results in the New Zealand sample closely resembled those observed in the U.S. sample. The overall conjunction fallacy error rate probability was only 5% for atheist targets (95% HPDI 0.01, 0.09), but 49% for religious targets (95% HPDI 0.37, 0.61). The probability of making the conjunction fallacy error was 12.0 times greater when the target was framed as religious.

Results of the full regression model for the extension are presented in Table 3. Our model showed a significant effect of target type such that the target coefficient falls within the confidence interval and is of the same sign (target = − 3.0, *SD* = 0.25, HPDI Low = − 4.18, HPDI High = − 2.00). As in the U.S. sample, there was also a significant interaction between target type and belief in God (target × belief = − 2.06, *SD* = 0.54, HPDI Low = − 3.17, HPDI High = − 1.06) such that higher levels of belief in God predicted greater conjunction fallacy rates for religious targets (see Fig. 1, top right panel).

**Table 3:** Full Model Summary for the Serial Helper Condition (New Zealand, Study 2).

Fixed effects	Coefficient	SD	95% HPDI	
			2.5%	97.5%
Target: Religious vs. Atheist	− 3.00	0.56	− 4.18	− 2.00
Belief in God	0.86	0.26	0.36	1.38
Age	0.04	0.20	− 0.36	0.44
Gender	− 0.17	0.22	− 0.59	0.25
SSES	0.38	0.23	− 0.06	0.84
Target X Belief	− 2.06	0.54	− 3.17	− 1.06

Age, gender, subjective socioeconomic status (SSES) and participant belief in God were standardized. Religious vs. atheist target was coded: atheist = 1, religious = 0. The 95% HPDI illustrates uncertainty around posterior means and indexes the interval in which the 95% most credible estimates lie.

#### Target type (religious versus atheist) and the relative risk of the conjunction fallacy in the Serial Killer condition

The present results departed somewhat from those of Gervais et al.^19^. Whereas they observed a nonsignificant trend in their New Zealand sample, we found that, as in the U.S. sample of Study 1, the conjunction fallacy was significantly more likely to be committed when the target was framed as an atheist than as religious. The model predicted an overall conjunction fallacy error rate probability of 45% for atheist targets (95% HPDI 0.35, 0.89) compared to 27% for religious targets (95% HPDI 0.28, 0.37). The relative risk of making the conjunction fallacy error was nearly 0.6 times greater when the target was an atheist. The full model likewise showed a significant effect of target type (see Table 4). Again, departing somewhat from the results of the prior work of Gervais et al.^19^, but replicating the findings of Study 1 in the U.S. sample, there was a significant interaction between target type and belief in God, such that higher levels of belief in God predicted greater conjunction fallacy rates for atheist targets (see Fig. 1, bottom right panel).

**Table 4:** Full Model Summary for the Serial Killer Condition (New Zealand, Study 2).

			95% HPDI	
Fixed effect	Coefficient	SD	2.5%	97.5%
Target: Religious vs. Atheist	0.78	0.35	0.12	1.46
Belief in God	− 0.27	0.26	− 0.79	0.22
Age	0.21	0.18	− 0.12	0.56
Gender	− 0.001	0.17	− 0.34	0.34
SSES	0.40	0.17	0.06	0.74
Target X Belief	0.72	0.34	0.05	1.39

Age, gender, subjective socioeconomic status (SSES) and participant belief in God were standardized. Religious vs. atheist target was coded: atheist = 1, religious = 0. The 95% HPDI illustrates uncertainty around posterior means and indexes the interval in which the 95% most credible estimates lie.

## General discussion

Here, we replicated and extended the findings of Gervais et al.^19^ and found intuitive moral biases both against atheists and in favor of the faithful, consistent with research that suggests religion may bolster cooperation and trust among believers^9,11,23^. Indeed, although Gervais et al.^19^ highlight their findings as evidence of an extreme intuitive moral bias against atheists, the present data suggest that the intuitive moral prejudice against atheists may be driven by a positive conceptual link between faith and prosociality.

Diverging from the results of Gervais et al.^19^, we observed relatively modest yet significant interactions between target type (atheist or believer) and individual differences in belief in God in the serial killer conditions of both studies. However, as these interactions were relatively modest, the slopes approximately resemble those obtained in the original studies, and the effects are readily interpretable within the relevant theoretical model (i.e., more devout individuals appear to harbor stronger moral biases related to religiosity), they do not substantively contradict the overall pattern of the original findings of Gervais et al.^19^. The sizable interactions between target type and belief in God in the serial helper conditions appear to be of greater note. Religious individuals were far more likely to associate prosociality with the prospect that the target character also believed in God, a result which is consistent with the premise that religion enhances trust and cooperation among co-religionists.

These findings lend support to the hypothesis that culturally evolved beliefs in moralizing gods may have spurred cooperation at increasing societal scales, such that individuals encountering heretofore unknown persons might be more inclined to help and less inclined to exploit one another given shared supernatural beliefs related to the enforcement of prosocial behavioral norms^12^. Prior cross-cultural work has demonstrated that religionists are more likely to behave generously toward strangers to the extent that they believe their god(s) monitor and exact punishments upon moral transgressors^24^, although the evidence that religious individuals are more likely to engage in prosocial behavior is mixed^25^. Future work extending the present studies might manipulate not only whether the target character is a believer, but whether they believe in a punitively judgmental god versus a god who forgives and excuses moral infractions; the moralizing gods hypothesis predicts that the former would be intuitively conceptualized as more helpful (and less murderous) than the latter. Future work might also assess the extent to which self-enhancement, the tendency for religious individuals to rate themselves as superior to non-religious individuals, contributes to our results^26^. Consistent with a self-enhancement interpretation, individual differences in religious faith moderated the effect of the manipulations in both studies, such that highly religious participants were more [less] prone to commit the conjunction fallacy when the serial helper [killer] was framed as religious. However, additional, distinct psychological processes must be at play as well, as we also observed significant tendencies to commit the conjunction fallacy among participants who reported low levels of religious faith, in line with the original findings reported by Gervais et al.^19^.

In sum, we found evidence that religionists are conceptualized as morally good to a greater extent than are atheists conceptualized as morally bad, with comparable patterns observed in a predominantly religious society, the United States, and in a predominantly secular society, New Zealand. Notwithstanding the aforementioned moderation of these effects by individual differences in religiosity, even relatively nonreligious participants evinced these biases in both societies, suggesting that the conceptual associations are pervasive. Following the example set by Gervais et al.^19^, future work should attempt to replicate the intuitive association between faith and prosociality reported here in a larger and more diverse array of societies from around the globe.

### Supplementary Information

Supplementary Information.

## Data Availability

The study was pre-registered using the Open Science Framework (OSF). The pre-registration document, data, code, and materials are publicly accessible at https://osf.io/2jq35/.

## References

[1] Atran, S. *In Gods We Trust* (Oxford University Press, 2002).

[2] Johnson, D. God’s punishment and public goods. *Hum. Nat.* **16**(4), 410–446 (2005). 26189839 10.1007/s12110-005-1017-0

[3] Shariff, A. F., Norenzayan, A. & Henrich, J. The birth of high gods: How the cultural evolution of supernatural policing influenced the emergence of complex, cooperative human societies, paving the way for civilization. In *Evolution, Culture, and the Human Mind* (eds Schaller, M. *et al.*) 119–136 (Psychology Press, 2010).

[4] McKay, R. T., Efferson, C., Whitehouse, H. & Fehr, E. Wrath of God: Religious primes and punishment. *Proc. R. Soc. B* **278**(1713), 1858–1863 (2011). 21106588 10.1098/rspb.2010.2125 PMC3097833

[5] Fitouchi, L. & Singh, M. Supernatural punishment beliefs as cognitively compelling tools of social control. *Curr. Opin. Psychol.* **44**, 252–257 (2022). 34752999 10.1016/j.copsyc.2021.09.022

[6] Durkheim, E. *The Elementary Forms of the Religious Life* (Allen & Unwin, 1912).

[7] Rappaport, R. *Ritual and Religion in the Making of Humanity* (Cambridge University Press, 1999).

[8] Atran, S. *Talking to the Enemy: Faith, Brotherhood, and the (un)Making of Terrorists* (HarperCollins, 2010).

[9] Graham, J. & Haidt, J. Beyond beliefs: Religions bind individuals into moral communities. *Pers. Soc. Psychol. Rev.* **14**(1), 140–150 (2010). 20089848 10.1177/1088868309353415

[10] Hall, D. L., Cohen, A. B., Meyer, K. K., Varley, A. H. & Brewer, G. A. Costly signaling increases trust, even across religious affiliations. *Psychol. Sci.* **26**(9), 1368–1376 (2015). 26187247 10.1177/0956797615576473

[11] Tan, J. H. & Vogel, C. Religion and trust: An experimental study. *J. Econ. Psychol.* **29**(6), 832–848 (2008). 10.1016/j.joep.2008.03.002

[12] Norenzayan, A. *et al.* The cultural evolution of prosocial religions. *Behav. Brain Sci.*10.1017/S0140525X14001356 (2016). 26785995 10.1017/S0140525X14001356

[13] Richerson, P. *et al.* Cultural group selection plays an essential role in explaining human cooperation: A sketch of the evidence. *Behav. Brain Sci.* **39**, E30. 10.1017/S0140525X1400106X (2016). 25347943 10.1017/S0140525X1400106X

[14] Gervais, W. M. Everything is permitted? People intuitively judge immorality as representative of atheists. *PloS ONE* **9**(4), e92302 (2014). 24717972 10.1371/journal.pone.0092302 PMC3981659

[15] Gervais, W. M., Shariff, A. F. & Norenzayan, A. Do you believe in atheists? Distrust is central to anti-atheist prejudice. *J. Pers. Soc. Psychol.* **101**(6), 1189 (2011). 22059841 10.1037/a0025882

[16] Brown-Iannuzzi, J. L., McKee, S. & Gervais, W. M. Atheist horns and religious halos: Mental representations of atheists and theists. *J. Exp. Psychol.: General* **147**(2), 292 (2018). 10.1037/xge0000376 29154618

[17] Brown, M. Preliminary evidence for an aversion to atheists in long-term mating domains in the Southern United States. *J. Soc. Pers. Relatsh.* **39**(3), 711–733 (2022). 10.1177/02654075211045051

[18] Pew Research Center. (2019). Available at: https://www.pewresearch.org/religion/2019/07/23/what-americans-know-about-religion/

[19] Gervais, W. M. *et al.**Nat. Hum. Behav.* **1**(8), 0151 (2017). 10.1038/s41562-017-0151

[20] Tversky, A. & Kahneman, D. Extensional versus intuitive reasoning—The conjunction fallacy in probability judgment. *Psychol. Rev.* **90**, 293–315 (1983). 10.1037/0033-295X.90.4.293 PMC4658440 26635712

[21] van Zwet, E. W. & Goodman, S. N. How large should the next study be? Predictive power and sample size requirements for replication studies. *Stat. Med.* **41**(16), 3090–3101 (2022). 35396714 10.1002/sim.9406 PMC9325423

[22] Adler, N. E., Epel, E. S., Castellazzo, G. & Ickovics, J. R. Relationship of subjective and objective social status with psychological and physiological functioning: Preliminary data in healthy white women. *Health Psychol.* **19**, 586–592 (2000). 11129362 10.1037/0278-6133.19.6.586

[23] Moon, J. W., Krems, J. A. & Cohen, A. B. Religious people are trusted because they are viewed as slow life-history strategists. *Psychol. Sci.* **29**(6), 947–960 (2018). 29590005 10.1177/0956797617753606

[24] Lang, M. *et al.* Moralizing gods, impartiality and religious parochialism across 15 societies. *Proc. R. Soc. B* **286**(1898), 20190202 (2019). 30836871 10.1098/rspb.2019.0202 PMC6458319

[25] Bendixen, T. *et al.* Gods are watching and so what? Moralistic supernatural punishment across 15 cultures. *Evol. Hum. Sci.* **5**, 1–30 (2023). 10.1017/ehs.2023.15 PMC10426076 37587943

[26] Sedikides, C. & Gebauer, J. E. Do religious people self-enhance?. *Curr. Opin. Psychol.* **40**, 29–33 (2021). 32892032 10.1016/j.copsyc.2020.08.002

