# Supplementary material for: Intuitive moral bias favors the religiously faithful
Source: Sci Rep. 2024 Aug 7;14:18291. doi: 10.1038/s41598-024-67960-4 (PMC11306559; doi:10.1038/s41598-024-67960-4)
Supplement: Supplementary file 1 — Supplementary Information. [file 41598_2024_67960_MOESM1_ESM.docx]

**Supplemental Online Materials**

**to accompany**

**Intuitive Moral Bias Favors the Religiously Faithful: Evidence from Two Societies**

**Table of Contents**

**Supplemental tables and analyses**

*Analyses Including Political Orientation*

- **Table S1.** Full Model Summary of the Effects of Target, Belief in God, Political Orientation, Age, Gender, and Subjective Social Status in the Serial Helper Condition (U.S., Study 1)
- **Table S2.** Full Model Summary of the Effects of Target, Belief in God, Political Orientation, Age, Gender, and Subjective Social Status in the Serial Killer Condition (U.S., Study 1)

*Analyses Combining Helper and Killer Conditions*

- **Table S3.** Full Model Summary of the Effects of Target, Helper/Killer Condition, Belief in God, Age, Gender, and Subjective Social Status (U.S., Study 1)
- **Table S4.** Full Model Summary of the Effects of Target, Helper/Killer Condition, Belief in God, Age, Gender, and Subjective Social Status (N.Z., Study 2)

*Analyses Combining Helper and Killer Conditions, Omitting Demographic Variables*

- **Table S5.** Full Model Summary of the Effects of Target, Helper/Killer Condition and Belief in God (U.S., Study 1)
- **Table S6.** Full Model Summary of the Effects of Target, Helper/Killer Condition and Belief in God (N.Z., Study 2)

*Analyses Including Feelings of Spiritual Connection to God*

- **Table S7.** Full Model Summary of the Effects of Target, Spiritual Connection, Age, Gender, and Subjective Social Status in the Serial Killer Condition (U.S., Study 1)
- **Table S8.** Full Model Summary of the Effects of Target, Spiritual Connection, Age, Gender, and Subjective Social Status in the Serial Helper Condition (U.S., Study 1)
- **Table S9.** Full Model Summary of the Effects of Target, Spiritual Connection, Age, Gender, and Subjective Social Status in the Serial Killer Condition (N.Z., Study 2)
- **Table S10.** Full Model Summary of the Effects of Target, Spiritual Connection, Age, Gender, and Subjective Social Status in the Serial Helper Condition (N.Z., Study 2)

**Study Materials**

The pre-registration, study materials, datasets, and code are available at <https://osf.io/2jq35/>

**Analyses Including Political Orientation**

| Table S1  *Full Model Summary of the Effects of Target, Belief in God, Political Orientation, Age, Gender, and Subjective Social Status in the Serial Helper Condition (U.S., Study 1)* | | | | |  |  |
| --- | --- | --- | --- | --- | --- | --- |
|  |  |  | 95% HPDI | |  | |
| Fixed effects | Coefficient | *SD* | 2.5% | 97.5% |  | |
| Target:Religious vs. Atheist | -2.04 | 0.28 | -2.63 | -1.53 |  | |
| Belief in God | 0.60 | 0.20 | 0.21 | 0.99 |  | |
| Age | 0.10 | 0.16 | -0.20 | 0.41 |  | |
| Gender | -0.11 | 0.15 | -0.41 | 0.18 |  | |
| SSES | 0.14 | 0.15 | -0.16 | 0.44 |  | |
| Political orientation | 0.67 | 0.21 | 0.26 | 1.10 |  | |
| Target X Belief | -1.92 | 0.53 | -3.02 | -0.96 |  | |
| Target X Politics | -1.06 | 0.42 | -1.92 | -0.26 |  | |

*Note.* Age, gender, subjective socioeconomic status (SSES) and participant belief in God were standardized. Religious vs. atheist target was coded: atheist = 1, religious = 0. The 95% highest posterior density intervals (HPDI) illustrate uncertainty around posterior means, and indexes the interval in which the 95% most credible estimates lie.

Political orientation was assessed using a modification of Dodd et al.’s (2012) version of Wilson and Patterson’s (1968) issues index. Participants were asked to indicate whether they agree, disagree, or are uncertain about various prominent issues in contemporary U.S. politics (e.g., abortion, tax rates, gun control). Agreement was scored as +1, disagreement as -1, and uncertainty as 0; liberal items were reverse scored and then the responses were composited, hence increasing positive values reflect greater conservatism (*M*_Political Orientation_ = -3.65, *SD =* 9.25).  The full model reveals a significant effect of target type (target = -2.04, sd = 0.28, HPDI Low = -2.63, HPDI High = -1.53), as well as a main effect of belief in God qualified by a significant interaction with target type (target x belief = -1.92, sd = 0.53, HPDI Low = -3.02, HPDI High = -0.96), such that participants who reported higher levels of belief in God were more likely to commit the conjunction fallacy rates in the religious target condition. There was a comparable effect of political orientation qualified by a significant interaction between target type and political orientation (target x politics = -1.06, sd = 0.42, HPDI Low = -1.92, HPDI High = -0.26), such that higher levels of conservatism predicted greater conjunction fallacy rates for religious targets.  Hence, political conservatism appeared to operate similarly to religious belief with respect to intuitively conceptualizing religious believers as prosocial.

| Table S2  *Full Model Summary of the Effects of Target, Belief in God, Political Orientation, Age, Gender, and Subjective Social Status in the Serial Killer Condition (U.S., Study 1)* | | | | |  |  |
| --- | --- | --- | --- | --- | --- | --- |
|  |  |  | 95% HPDI | |  | |
| Fixed effects | Coefficient | *SD* | 2.5% | 97.5% |  | |
| Target:Religious vs. Atheist | 2.15 | 0.28 | 1.61 | 2.71 |  | |
| Belief in God | 0.19 | 0.21 | -0.20 | 0.61 |  | |
| Age | 0.03 | 0.13 | -0.24 | 0.30 |  | |
| Gender | -0.05 | 0.14 | -0.31 | 0.22 |  | |
| SSES | -0.06 | 0.14 | -0.33 | 0.21 |  | |
| Political orientation | -0.16 | 0.22 | -0.60 | 0.28 |  | |
| Target X Belief | 0.29 | 0.28 | -0.27 | 0.85 |  | |
| Target X Politics | 0.82 | 0.29 | 0.25 | 1.41 |  | |

*Note.* Age, gender, subjective socioeconomic status (SSES) and participant belief in God were standardized. Religious vs. atheist target was coded: atheist = 1, religious = 0. The 95% highest posterior density intervals (HPDI) illustrate uncertainty around posterior means, and indexes the interval in which the 95% most credible estimates lie.

The full model reveals a significant effect of target type (target = 2.15, sd = 0.28, HPDI Low = 1.61, HPDI High = 2.71), but no main effect of belief in God nor an interaction between target type and belief in God. However, there was a significant interaction between target type and political orientation such that participants who reported a higher levels of political conservatism were more likely to commit the conjunction fallacy in the atheist target condition (target x politics = 0.82, sd = 0.29, HPDI Low = 0.25, HPDI High = 1.41).

**Analyses Combining Helper and Killer conditions**

| Table S3  *Full Model Summary of the Effects of Target, Helper/Killer Condition, Belief in God, Age, Gender, and Subjective Social Status (U.S., Study 1)* | | | | |  |  |
| --- | --- | --- | --- | --- | --- | --- |
|  |  |  | 95% HPDI | |  | |
| Fixed effects | Coefficient | *SD* | 2.5% | 97.5% |  | |
| Target:Religious vs. Atheist | -2.83 | 0.32 | -3.47 | -2.25 |  | |
| Condition: Killer vs Helper | -1.54 | 0.22 | -1.99 | -1.12 |  | |
| Belief in God | 0.82 | 0.16 | 0.52 | 1.14 |  | |
| Age | 0.05 | 0.09 | -0.13 | 0.23 |  | |
| Gender | -0.09 | 0.09 | -0.28 | 0.09 |  | |
| SSES | -0.01 | 0.09 | -0.18 | 0.17 |  | |
| Target X Belief | -1.46 | 0.31 | -2.11 | -0.86 |  | |
| Target X Condition | 4.51 | 0.38 | 3.78 | 5.26 |  | |
| Belief X Condition | -0.57 | 0.23 | -1.03 | -0.13 |  | |
| Target X Condition X Belief | 1.98 | 0.38 | 1.26 | 2.74 |  | |

*Note.* Age, gender, subjective socioeconomic status (SSES) and participant belief in God were standardized. Religious vs. atheist target was coded: atheist = 1, religious = 0. Experimental condition was coded: serial killer = 1, serial helper = 0. The 95% highest posterior density intervals (HPDI) illustrate uncertainty around posterior means, and indexes the interval in which the 95% most credible estimates lie.

The full model reveals a significant effect of target type (target = -2.83, sd = 0.32, HPDI Low = -3.47, HPDI High = -2.25), and a main effect of belief in God qualified by a significant interaction with target type (target x belief = -1.46, sd = 0.31, HPDI Low = -2.11, HPDI High = -0.86), such that participants who reported higher levels of belief in God were more likely to commit the conjunction fallacy rates with a religious target in the serial helper condition. On the other hand, the interaction effect of belief in God qualified by a significant interaction with both target type and condition (target x condition x belief = 1.98, sd = 0.38, HPDI Low = 1.26, HPDI High = 2.74), such that participants who reported higher levels of belief in God were more likely to commit the conjunction fallacy rates with an atheist target in the serial killer condition.

| Table S4  *Full Model Summary of the Effects of Target, Helper/Killer Condition, Belief in God, Age, Gender, and Subjective Social Status (N.Z., Study 2)* | | | | |  |  |
| --- | --- | --- | --- | --- | --- | --- |
|  |  |  | 95% HPDI | |  | |
| Fixed effects | Coefficient | *SD* | 2.5% | 97.5% |  | |
| Target:Religious vs. Atheist | -2.17 | 0.40 | -2.96 | -1.41 |  | |
| Condition: Killer vs Helper | -0.58 | 0.32 | -1.22 | 0.04 |  | |
| Belief in God | 0.72 | 0.23 | 0.26 | 1.19 |  | |
| Age | 0.12 | 0.13 | -0.14 | 0.37 |  | |
| Gender | -0.05 | 0.13 | -0.31 | 0.20 |  | |
| SSES | 0.35 | 0.13 | 0.10 | 0.61 |  | |
| Target X Belief | -1.35 | 0.39 | -2.13 | -0.59 |  | |
| Target X Condition | 2.63 | 0.48 | 1.69 | 3.56 |  | |
| Belief X Condition | -0.83 | 0.32 | -1.47 | -0.22 |  | |
| Target X Condition X Belief | 1.84 | 0.47 | 0.90 | 2.73 |  | |

*Note.* Age, gender, subjective socioeconomic status (SSES) and participant belief in God were standardized. Religious vs. atheist target was coded: atheist = 1, religious = 0. Experimental condition was coded: serial killer = 1, serial helper = 0. The 95% highest posterior density intervals (HPDI) illustrate uncertainty around posterior means, and indexes the interval in which the 95% most credible estimates lie.

The full model reveals a significant effect of target type (target = -2.17, sd = 0.40, HPDI Low = -2.96, HPDI High = -1.41, as well as a main effect of belief in God qualified by a significant interaction with target type (target x belief = -1.35, sd = 0.39, HPDI Low = -2.13, HPDI High = -0.59), such that participants who reported higher levels of belief in God were more likely to commit the conjunction fallacy rates with a religious target in the serial helper condition. On the other hand, the interaction effect of belief in God qualified by a significant interaction with both target type and condition (target x condition x belief = 1.84, sd = 0.47, HPDI Low = 0.90, HPDI High = 2.73), such that participants who reported higher levels of belief in God were more likely to commit the conjunction fallacy rates with an atheist target in the serial killer condition.

**Analyses combining Helper and Killer Conditions, Omitting Demographic Variables**

| Table S5  *Full Model Summary of the Effects of Target, Helper/Killer Condition and Belief in God (U.S., Study 1)* | | | | |  |  |
| --- | --- | --- | --- | --- | --- | --- |
|  |  |  | 95% HPDI | |  | |
| Fixed effects | Coefficient | *SD* | 2.5% | 97.5% |  | |
| Target:Religious vs. Atheist | -2.80 | 0.32 | -3.45 | -2.20 |  | |
| Condition: Killer vs Helper | -1.54 | 0.23 | -2.00 | -1.11 |  | |
| Belief in God | 0.81 | 0.15 | 0.51 | 1.11 |  | |
| Target X Belief | -1.46 | 0.30 | -2.06 | -0.88 |  | |
| Target X Condition | 4.49 | 0.37 | 3.78 | 5.22 |  | |
| Belief X Condition | -0.58 | 0.23 | -1.01 | -0.13 |  | |
| Target X Condition X Belief | 2.00 | 0.38 | 1.26 | 2.75 |  | |

*Note.* Age, gender, subjective socioeconomic status (SSES) and participant belief in God were standardized. Religious vs. atheist target was coded: atheist = 1, religious = 0. Experimental condition was coded: serial killer = 1, serial helper = 0. The 95% highest posterior density intervals (HPDI) illustrate uncertainty around posterior means, and indexes the interval in which the 95% most credible estimates lie.

The full model reveals a significant effect of target type (target = -2.80, sd = 0.32, HPDI Low = -3.45, HPDI High = -2.20), and a main effect of belief in God qualified by a significant interaction with target type (target x belief = -1.46, sd = 0.30, HPDI Low = -2.06, HPDI High = -0.88), such that participants who reported higher levels of belief in God were more likely to commit the conjunction fallacy rates with a religious target in the serial helper condition. On the other hand, the interaction effect of belief in God qualified by a significant interaction with both target type and condition (target x condition x belief = 2.00, sd = 0.38, HPDI Low = 1.26, HPDI High = 2.75), such that participants who reported higher levels of belief in God were more likely to commit the conjunction fallacy rates with an atheist target in the serial killer condition.

| Table S6  *Full Model Summary of the Effects of Target, Helper/Killer Condition and Belief in God (N.Z., Study 2)* | | | | |  |  |
| --- | --- | --- | --- | --- | --- | --- |
|  |  |  | 95% HPDI | |  | |
| Fixed effects | Coefficient | *SD* | 2.5% | 97.5% |  | |
| Target:Religious vs. Atheist | -2.01 | 0.39 | -2.78 | -1.25 |  | |
| Condition: Killer vs Helper | -0.49 | 0.31 | -1.10 | 0.11 |  | |
| Belief in God | 0.73 | 0.23 | 0.28 | 1.19 |  | |
| Target X Belief | -1.31 | 0.39 | -2.07 | -0.53 |  | |
| Target X Condition | 2.49 | 0.46 | 1.62 | 3.38 |  | |
| Belief X Condition | -0.85 | 0.32 | -1.49 | -0.22 |  | |
| Target X Condition X Belief | 1.79 | 0.47 | 0.85 | 2.69 |  | |

*Note.* Age, gender, subjective socioeconomic status (SSES) and participant belief in God were standardized. Religious vs. atheist target was coded: atheist = 1, religious = 0. Experimental condition was coded: serial killer = 1, serial helper = 0. The 95% highest posterior density intervals (HPDI) illustrate uncertainty around posterior means, and indexes the interval in which the 95% most credible estimates lie.

The full model reveals a significant effect of target type (target = -2.01, sd = 0.39, HPDI Low = -2.78, HPDI High = -1.25, as well as a main effect of belief in God qualified by a significant interaction with target type (target x belief = -1.31, sd = 0.39, HPDI Low = -2.07, HPDI High = -0.53), such that participants who reported higher levels of belief in God were more likely to commit the conjunction fallacy rates with a religious target in the serial helper condition. On the other hand, the interaction effect of belief in God qualified by a significant interaction with both target type and condition (target x condition x belief = 1.79, sd = 0.47, HPDI Low = 0.85, HPDI High = 2.69), such that participants who reported higher levels of belief in God were more likely to commit the conjunction fallacy rates with an atheist target in the serial killer condition.

**Analyses Including Feelings of Spiritual Connection to God**

Feelings of spiritual connection to God were measured using a modified version of the Inclusion of the Other in the Self scale composed of seven pairs of circles ranging from nonoverlapping to almost entirely overlapping (Aron, Aron, & Smollan, 1992) This closeness measure was framed as distinct from trait religious belief because individuals may feel a spiritual connection to God while simultaneously lacking a firm belief in God.

| Table S7  *Full Model Summary of the Effects of Target, Spiritual Connection, Age, Gender, and Subjective Social Status in the Serial Killer Condition (U.S., Study 1)* | | | | |  |  |
| --- | --- | --- | --- | --- | --- | --- |
|  |  |  | 95% HPDI | |  | |
| Fixed effects | Coefficient | *SD* | 2.5% | 97.5% |  | |
| Target:Religious vs. Atheist | 2.08 | 0.25 | 1.58 | 2.56 |  | |
| Spiritual | 0.26 | 0.17 | -0.07 | 0.60 |  | |
| Age | 0.02 | 0.13 | -0.23 | 0.27 |  | |
| Gender | -0.07 | 0.13 | -0.32 | 0.19 |  | |
| SSES | -0.09 | 0.13 | -0.34 | 0.16 |  | |
| Target X Spiritual | 0.30 | 0.24 | -0.16 | 0.78 |  | |

*Note.* Age, gender, subjective socioeconomic status (SSES) and participant belief in God were standardized. Religious vs. atheist target was coded: atheist = 1, religious = 0. The 95% highest posterior density intervals (HPDI) illustrate uncertainty around posterior means, and indexes the interval in which the 95% most credible estimates lie.

The full model reveals a significant effect of target type (target = 2.08, sd = 0.25, HPDI Low = 1.58, HPDI High = 2.56). There was no effect of spiritual connection, nor interaction between target type and spiritual connection to God (target x spiritual  = 0.30, sd = 0.24, HPDI Low = -0.16, HPDI High = 0.78).

| Table S8  *Full Model Summary of the Effects of Target, Spiritual Connection, Age, Gender, and Subjective Social Status in the Serial Helper Condition (U.S., Study 1)* | | | | |  |  |
| --- | --- | --- | --- | --- | --- | --- |
|  |  |  | 95% HPDI | |  | |
| Fixed effects | Coefficient | *SD* | 2.5% | 97.5% |  | |
| Target:Religious vs. Atheist | -3.55 | 0.44 | -4.50 | -2.76 |  | |
| Spiritual | 0.63 | 0.16 | 0.32 | 0.94 |  | |
| Age | 0.19 | 0.14 | -0.09 | 0.47 |  | |
| Gender | -0.07 | 0.14 | -0.35 | 0.20 |  | |
| SSES | 0.09 | 0.14 | -0.18 | 0.37 |  | |
| Target X Spiritual | -1.74 | 0.44 | -2.66 | -0.94 |  | |

*Note.* Age, gender, subjective socioeconomic status (SSES) and participant belief in God were standardized. Religious vs. atheist target was coded: atheist = 1, religious = 0. The 95% highest posterior density intervals (HPDI) illustrate uncertainty around posterior means, and indexes the interval in which the 95% most credible estimates lie.

The full model reveals a significant effect of target type (target = 3.55, sd = 0.44, HPDI Low = -4.50, HPDI High = 2.76). There was a significant interaction between target type and spiritual connection to God (target x spiritual  = -1.74, sd = 0.44, HPDI Low = -2.66, HPDI High = -0.94), such that higher levels of spirituality predicted greater conjunction fallacy rates for religious targets, comparable to the pattern observed for Belief in God (see main text).

| Table S9  *Full Model Summary of the Effects of Target, Spiritual Connection, Age, Gender, and Subjective Social Status in the Serial Killer Condition (N.Z., Study 2)* | | | | |  |  |
| --- | --- | --- | --- | --- | --- | --- |
|  |  |  | 95% HPDI | |  | |
| Fixed effects | Coefficient | *SD* | 2.5% | 97.5% |  | |
| Target:Religious vs. Atheist | 0.86 | 0.36 | 0.15 | 1.60 |  | |
| Spiritual | -0.62 | 0.30 | -1.24 | -0.07 |  | |
| Age | 0.18 | 0.17 | -0.17 | 0.52 |  | |
| Gender | -0.004 | 0.17 | -0.35 | 0.33 |  | |
| SSES | 0.41 | 0.18 | 0.06 | 0.76 |  | |
| Target X Spiritual | 0.96 | 0.37 | 0.28 | 1.70 |  | |

*Note.* Age, gender, subjective socioeconomic status (SSES) and participant belief in God were standardized. Religious vs. atheist target was coded: atheist = 1, religious = 0. The 95% highest posterior density intervals (HPDI) illustrate uncertainty around posterior means, and indexes the interval in which the 95% most credible estimates lie.

The full model reveals a significant effect of target type (target = 0.86, sd = 0.36, HPDI Low = 0.15, HPDI High = 1.60). There was also a significant interaction between target type and spiritual connection to God (target x spiritual  = 0.96, sd = 0.37, HPDI Low = 0.28, HPDI High = 1.70), such that higher levels of spirituality predicted greater conjunction fallacy rates for atheist targets.

| Table S10  *Full Model Summary of the Effects of Target, Spiritual Connection, Age, Gender, and Subjective Social Status in the Serial Helper Condition (N.Z., Study 2)* | | | | |  |  |
| --- | --- | --- | --- | --- | --- | --- |
|  |  |  | 95% HPDI | |  | |
| Fixed effects | Coefficient | *SD* | 2.5% | 97.5% |  | |
| Target:Religious vs. Atheist | -2.87 | 0.52 | -3.95 | -1.92 |  | |
| Spiritual | 0.45 | 0.25 | -0.04 | 0.95 |  | |
| Age | 0.08 | 0.20 | -0.33 | 0.47 |  | |
| Gender | -0.24 | 0.20 | -0.64 | 0.15 |  | |
| SSES | 0.33 | 0.20 | -0.05 | 0.73 |  | |
| Target X Spiritual | -1.46 | 0.55 | -2.64 | -0.45 |  | |

*Note.* Age, gender, subjective socioeconomic status (SSES) and participant belief in God were standardized. Religious vs. atheist target was coded: atheist = 1, religious = 0. The 95% highest posterior density intervals (HPDI) illustrate uncertainty around posterior means, and indexes the interval in which the 95% most credible estimates lie.

The full model reveals a significant effect of target type (target = 2.87, sd = 0.52, HPDI Low = -3.95, HPDI High = -1.92). There was a significant interaction between target type and spiritual connection to God (target x spiritual  = -1.46, sd = 0.55, HPDI Low = -2.64, HPDI High = -0.45), such that higher levels of spirituality predicted greater conjunction fallacy rates for religious targets.

**Study Materials**

**Q1 (Serial Killer—Belief)**When a man was young, he began inflicting harm on animals. It started with just pulling the wings off flies, but eventually progressed to torturing stray cats and other animals in his neighborhood.
 
As an adult, the man found that he did not get much thrill from harming animals, so he began hurting people instead. He has killed 5 homeless people that he abducted from poor neighborhoods in his home city. Their dismembered bodies are currently buried in his basement.

 Which is more probable?

o The man is a teacher.

o The man is a teacher and believes in God. 

**Q1 (Serial Killer—No Belief)**

When a man was young, he began inflicting harm on animals. It started with just pulling the wings off flies, but eventually progressed to torturing stray cats and other animals in his neighborhood.
 
As an adult, the man found that he did not get much thrill from harming animals, so he began hurting people instead. He has killed 5 homeless people that he abducted from poor neighborhoods in his home city. Their dismembered bodies are currently buried in his basement.

o The man is a teacher.

o The man is a teacher and does not believe in God.

**Q1 (Serial Helper—Belief)**

When a man was young, he began helping stray animals. It started with just putting out water for birds, but eventually progressed to fostering stray cats and other animals in his neighborhood.
 
As an adult, the man felt inspired to help people in need as well. Each week he visits poor neighborhoods in his city and offers food and clothes to homeless people. Sometimes when the weather is very cold he offers homeless families a place to stay in a spare room in his house.

 Which is more probable?

o The man is a teacher.

o The man is a teacher and believes in God.

**Q1 (Serial Helper—No Belief)**

When a man was young, he began helping stray animals. It started with just putting out water for birds, but eventually progressed to fostering stray cats and other animals in his neighborhood.
 
As an adult, the man felt inspired to help people in need as well. Each week he visits poor neighborhoods in his city and offers food and clothes to homeless people. Sometimes when the weather is very cold he offers homeless families a place to stay in a spare room in his house.

Which is more probable?

o The man is a teacher.

o The man is a teacher and does not believe in God.

**Q2 Puzzles**

In a lake, there is a patch of lily pads. Every day, the patch doubles in size. If it takes 48 days for the patch to cover the entire lake, how long would it take for the patch to cover half of the lake?

________________________________________________________________

If it takes 5 machines 5 minutes to make 5 widgets, how long would it take 100 machines to make 100 widgets?

________________________________________________________________

A bat and a ball cost $1.10 in total. The bast costs $1.00 more than the ball. How much does the ball cost?

________________________________________________________________

**Q3 Attention Check**

Here is a different type of question. SKIP THIS QUESTION, PLEASE. It is only included to ensure that you are paying attention and reading directions. Do not leave an answer for the question about US presidents.

Who is the current President of the United States of America?

o Donald J. Trump

o Mitt Romney

o Steve Perry

o George Washington

**Q4 Suspicion**

 What do you think this study is mainly about so far?

o Stereotyping and prejudice

o Logic and reasoning

o Language fluency

o Emotion perception

o Memory

**Demographic Questions**

**Q5** How old are you?

________________________________________________________________

**Q6** What is your gender?

o Male

o Female

o Other

**Q7** What country do you live in?

o United States

o Germany

o New Zealand

o Mexico

o United Kingdom

o India

o Other

**Q8** What is your religious affiliation?

o Christian (Catholic)

o Christian (Baptist)

o Christian (Other)

o Hindu

o Buddhist

o Muslim

o Jewish

o Sikh

o None

o Atheist

o Agnostic

o Other (Please specify)   ________________________________________________

**Q9 Perceived SES**

We are interested in how you perceive your life. Think of a ladder representing where people stand in (the U.S. or New Zealand). At the top of the ladder are people who are the best off--those who have the most money, the most education, and the most respected jobs. At the bottom are the people who are worst off--who have the least money, the least education, and the least respected jobs or no job. The higher up you are on this ladder, the closer you are to the top; the lower you are, the closer you are to people at the very bottom. Imagine this rating scale represents the ladder. Where would you place yourself, relative to the other people in (the U.S. or New Zealand? A response of '0' represents the bottom of the ladder and a response of '10' represents the top of the ladder.

o 10

o 9

o 8

o 7

o 6

o 5

o 4

o 3

o 2

o 1

o 0

**Q10 Belief in God**

Religious Scale How strongly do you believe in God or gods (from 0-100)? To clarify, if you are certain that God (or gods) does not exist, please put "0" and if you are certain that God (or gods) does exist, then put "100).

________________________________________________________________

**Q11(US Version)** How would you describe your race/ethnicity?

o White

o Hispanic/Latinx

o Black/African American

o American Indian/Alaskan Native

o Asian

o Native Hawaiian/Pacific Islander

o Mixed

o Other:   ________________________________________________

**Q11(NZ Version)** How would you describe your race/ethnicity?

o New Zealand European

o Maori

o Samoan

o Cook Islands Maori

o Tongan

o Niuean

o Chinese

o Indian

o Other:   ________________________________________________

**Q12** **Spiritual Connection**

People feel different levels of connection with God or a Higher Power. Some people may have firm belief, but not feel a strong connection, while other people may have less firm belief, but still feel a strong connection.

Imagine that the pairs of circles below represent you and God or a Higher Power. Please select the option below that best describes **how closely connected** you feel with God or a Higher Power:

 
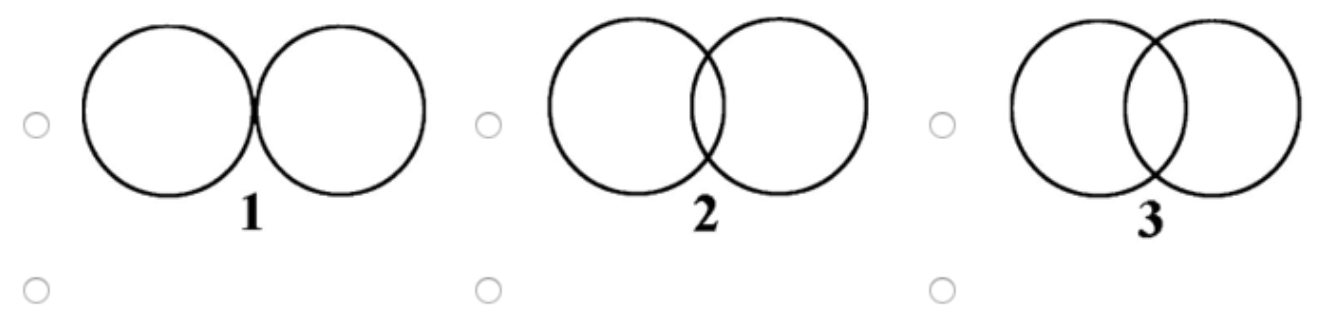


**
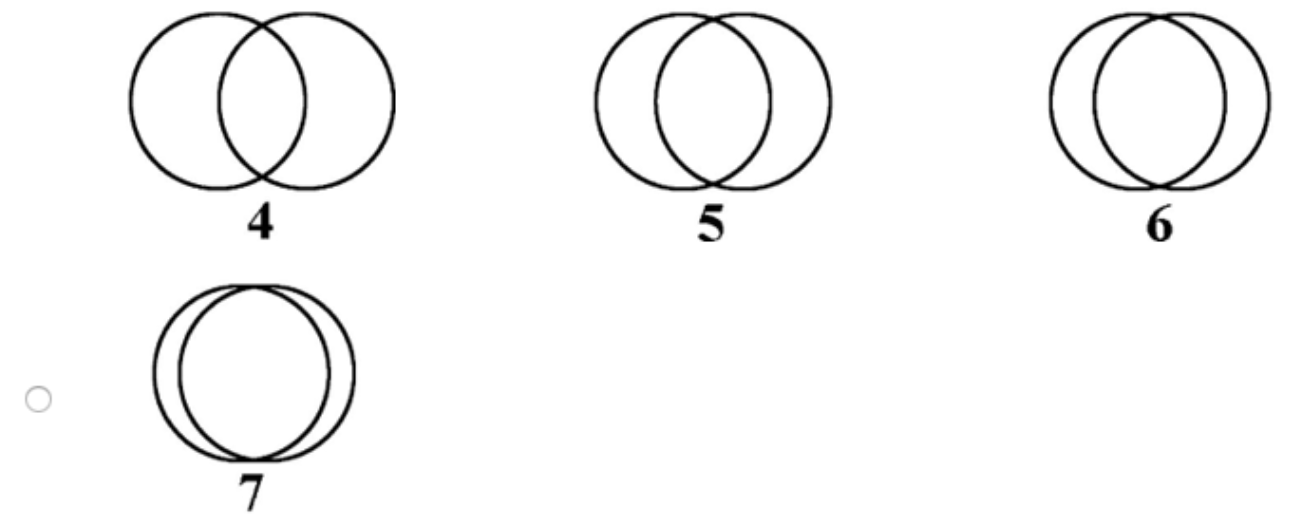
**

**Please rate the extent to which you AGREE with the following statements about your religious beliefs:**

**Q13** I believe in a personal God or a Higher Power.

|  | 1 | 2 | 3 | 4 | 5 | 6 | 7 | 8 | 9 |  |
| --- | --- | --- | --- | --- | --- | --- | --- | --- | --- | --- |
| *Not at all* | o | o | o | o | o | o | o | o | o | *Extremely* |

**Q14** When I am in trouble, I find myself wanting to ask God or a Higher Power for help.

|  | 1 | 2 | 3 | 4 | 5 | 6 | 7 | 8 | 9 |  |
| --- | --- | --- | --- | --- | --- | --- | --- | --- | --- | --- |
| *Not at all* | o | o | o | o | o | o | o | o | o | *Extremely* |

**Q15** When people pray, they are only talking to themselves.

|  | 1 | 2 | 3 | 4 | 5 | 6 | 7 | 8 | 9 |  |
| --- | --- | --- | --- | --- | --- | --- | --- | --- | --- | --- |
| *Not at all* | o | o | o | o | o | o | o | o | o | *Extremely* |

**Q16** What is the highest degree of education you have completed?

o Some high school

o Completed high school or equivalent

o Some university/college

o Completed university/college

o Some postgraduate work

o Completed a postgraduate degree

**Q17** How much do you agree with the following statement?

In today's world, legal and educational systems are more important than religious belief to make sure that people do not harm one another.

o Strongly disagree

o Disagree

o Somewhat disagree

o Neither agree nor disagree

o Somewhat agree

o Somewhat  agree

o Agree

o Strongly agree

**Q18** How much do you agree with the following statement?

In today's world, legal and educational systems are more important than religious belief to make sure that people help one another.

o Strongly disagree

o Disagree

o Somewhat disagree

o Neither agree nor disagree

o Somewhat agree

o Somewhat  agree

o Agree

o Strongly agree

**Q19 Politics  (US only)**

*Please indicate whether you AGREE, DISAGREE, or are UNCERTAIN, with regard to each topic listed below:*

Agree           Disagree         Uncertain

Pacifism       
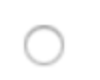

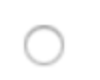

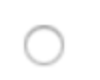


More restricted immigration               
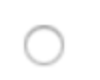

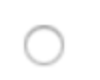

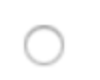


Death penalty                                      
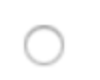

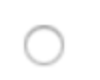

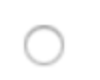


Harsh interrogation of                         
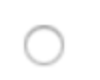

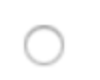

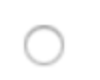


suspected terrorists

Military attacks of foreign                   
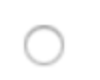

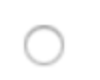

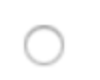


enemies

School prayer                                       
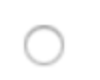

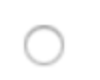

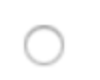


Socialism                                             
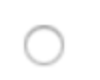

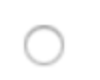

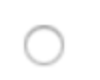


Same sex marriage                               
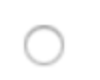

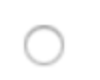

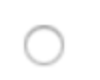


Abortion rights                                     
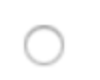

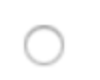

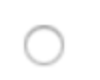


*Please indicate whether you AGREE, DISAGREE, or are UNCERTAIN, with regard to each topic listed below:*

Agree           Disagree         Uncertain

Military spending     
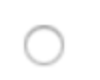

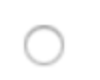

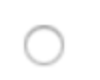


Warrentless searches                        
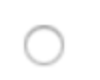

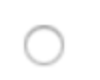

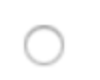


Drone strikes on terror suspects          
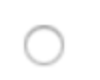

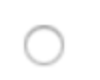

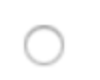


Harsh interrogation of                         
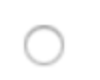

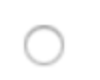

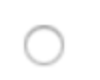


suspected terrorists

Obedience to authority                        
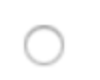

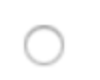

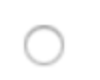


Compromise with enemies                  
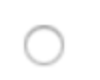

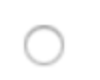

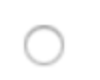


Literal existence of God                      
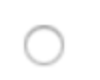

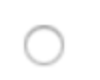

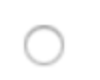


Welfare spending                                 
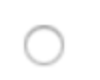

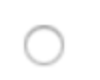

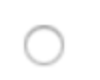


Tax cuts                                                
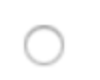

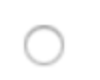

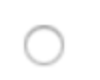


Reduce regulations on business           
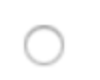

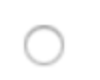

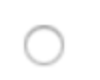


Recreational drug use                          
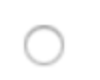

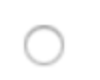

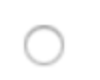


**References**

Aron, A., Aron, E. N., & Smollan, D. (1992). Inclusion of other in the self scale and the

structure of interpersonal closeness. *Journal of personality and social psychology*, *63*(4),

596.

Dodd, M. D., Balzer, A., Jacobs, C. M., Gruszczynski, M. W., Smith, K. B., & Hibbing, J. R.

(2012). The political left rolls with the good and the political right confronts the bad: connecting physiology and cognition to preferences. *Philosophical Transactions of the Royal Society B: Biological Sciences*, *367*(1589), 640-649.
